# Regional variation in hospitalization for stroke among Asians/Pacific Islanders in the United States: a nationwide retrospective cohort study

**DOI:** 10.1186/1471-2377-5-21

**Published:** 2005-11-10

**Authors:** Mai N Nguyen-Huynh, S Claiborne Johnston

**Affiliations:** 1 Department of Neurology, University of California San Francisco, 505 Parnassus Avenue, San Francisco, California 94143, USA; 2 Department of Epidemiology, University of California San Francisco, 505 Parnassus Avenue, San Francisco, California 94143, USA

## Abstract

**Background:**

In Asia, stroke incidence varies dramatically from country to country. Little is known about stroke incidence in Asians/Pacific Islanders in the US, where regional heterogeneity in Asian/Pacific Islander sub-populations is great. We sought to characterize both the national and regional incidences of first and recurrent hospitalized acute ischemic stroke, subarachnoid hemorrhage, and intracerebral hemorrhage in Asians/Pacific Islanders compared to non-Hispanic whites.

**Methods:**

We used the National Inpatient Sample of the 1997 Healthcare Cost and Utilization Project. It is a 20% stratified sample of hospitalizations to nonfederal hospitals in the US. National and regional projections were made using sampling weights specific for patients and hospitals. We identified stroke subtypes using previously validated *ICD-9 *codes. Age-adjusted incidence rates were calculated using the direct method with the US population in 2000 as the standard.

**Results:**

There were 169,386 stroke hospitalizations in the database. Nationally, compared to whites, Asians/Pacific Islanders were more likely to have subarachnoid hemorrhage (incidence rate ratio {RR} female: 1.53, 95% CI 1.41–1.65; male RR: 1.13, 95% CI 1.00–1.27) and intracerebral hemorrhage (female RR 1.29, 95% CI 1.22–1.36; male RR: 1.58, 95% CI 1.50–1.67). However, when examined by geographic regions, Asians/Pacific Islanders had higher incidence rates of subarachnoid hemorrhage and intracerebral hemorrhage predominantly in the West, and lower rates of stroke elsewhere.

**Conclusion:**

Stroke incidence varies 3-fold among Asians/Pacific Islanders residing in different US regions. Geographic variation is less dramatic in whites. Whether genetic or cultural differences are responsible for dramatic heterogeneity among Asian/Pacific Islander populations is unclear and deserves further study.

## Background

Stroke is a disease of major importance in Asia. Reported incidence rates vary dramatically but are generally higher than those of the US [1-3]. For example, reported incidence rates for overall stroke are 39% greater in Japan [4], 23% greater in Taiwan [5], and 81% greater in Northern China[6] compared to the US. It is the second leading cause of death in China, Korea and Taiwan; third in Japan and Singapore; sixth in the Philippines; and tenth in Thailand [3,7-9].

The incidence of stroke among Asians/Pacific Islanders (APIs) in the US is largely unstudied. The only major epidemiologic study on stroke in a sub-population of APIs was the Honolulu Heart Program [10]. A recent comparison of the 20-year incidence of hospitalized stroke between Japanese-American men in the Honolulu Heart Program and white men in the Framingham Study found a 40% excess of thromboembolic stroke in whites [11]. The incidence of hemorrhagic stroke was similar in the two cohorts. Another study reported a higher mortality rate from hemorrhagic stroke in APIs compared to whites in the US, and that deaths tended to occur at younger ages for some stroke types among APIs [12]. Some small studies have reported differences in the distribution of stroke subtypes between APIs and whites in the US [13,14]. APIs have constituted only a small proportion of prior population-based cohorts in the US.

Although race-ethnic disparities within the US has been a recent interest [15], the vast majority of studies have focused on African Americans and Hispanics [16,17], and few studies have described stroke in APIs in the US. API constitutes approximately 4% of the total US population and represents the fastest growing racial-ethnic group in the country. The Bureau of Census estimates that the number of Asians aged 25 years or older will increase 5-fold from 1995 to 2050 and that API will reach 10% of the US population by the year 2050 [18]. In 1998, the Department of Health and Human Services launched the Initiative to Eliminate Racial and Ethnic Disparities in Health with stroke identified as one of the six targeted conditions, and Healthy People 2010 prevention agenda[19] has the same objective.

Examining race-ethnic distributions of stroke incidence and mortality is required to define the burden of disease and to help guide the development of directed prevention programs, and may also be useful in identifying new genetic or environmental risk factors. We sought to characterize the incidence of hospitalized acute ischemic stroke, subarachnoid hemorrhage (SAH), and intracerebral hemorrhage (ICH) in APIs compared to non-Hispanic whites, using data representative of US hospitalizations in 1997. To assess whether stroke rates might differ in API sub-populations in the US, we also characterized regional variation.

## Methods

We used the Nationwide Inpatient Sample of the 1997 Healthcare Cost Utilization Project (HCUP), release 6, produced by the Agency for Healthcare Research and Quality [20]. The Nationwide Inpatient Sample (NIS) is a sample of US community hospitals, including nonfederal, short-term, general and specialty hospitals. It excludes all long-term hospitals, psychiatric hospitals, and alcoholism/chemical dependency treatment facilities. The NIS is based on a 20% stratified probability sample of hospitals with sampling probabilities proportional to the total number of US community hospitals in each stratum. The five stratification variables are geographic region (Northeast, Midwest, South, and West), ownership (government nonfederal, private not-for-profit, private investor-owned), location (urban vs. rural), teaching status (teaching vs. non-teaching), and size based on number of beds (small, medium and large). The database includes information typically available from discharge abstracts for 7,148,420 inpatient stays in 1,012 hospitals across 22 geographically dispersed states (AZ, CA, CO, CT, FL, GA, HI, IL, IA, KS, MD, MA, MO, NJ, NY, OR, PA, SC, TN, UT, WA, WI). Our study was approved by the Committee on Human Research at the University of California San Francisco.

### Incidence of hospitalized strokes

HCUP does not distinguish between first and recurrent stroke admissions; therefore, we report the incidence of first and recurrent stroke leading to hospitalization. We identified all patients with a primary diagnosis of stroke (International Classification of Diseases, Ninth Revision, [*ICD-9*] codes 430–434, 436). Stroke subtypes were identified from previously identified codes [21-23]: acute ischemic stroke (*ICD-9 *433.01, 433.11, 433.21, 433.31, 433.81, 433.91, 434.01, 434.11, 434.91, 436), SAH (*ICD-9 *430), and ICH (*ICD-9 *431). For standardization of stroke incidence, age groups were defined as 0–19 years, 20–29, 30–39, 40–49, 50–59, 60–64, 65–74, 75–84, and 85+. Gender was classified as male and female. Race-ethnicity was coded as non-Hispanic white, Black, Hispanic, API, Native American, and other, based on self-report.

Unadjusted incidence rates were calculated using the number of cases in the database, the appropriate discharge sampling weights specific for each age group, stroke type, and race-ethnicity, and with the 1997 non-Hispanic API or white populations based on Census Bureau data [24] as the denominators. Age-adjusted and gender-specific incidence rates were calculated by the direct method using the entire US population in 2000 as the standard [25]. The number of exposed individuals for each age category was calculated by dividing the 1997 US population in each age category by the discharge sampling weight for the corresponding age category. Subsequently, an event rate for each age category was calculated by dividing the number of cases identified in the database by the number of exposed individuals. Age adjusted rate equals to the sum of age-specific rates multiplied by the age-specific US population proportions. Standard errors for all age categories were computed, as well as an overall standard error for the age-adjusted incidence rate. A 95% confidence interval (CI) was equal to the adjusted rate ± 1.96 times the standard error.

Estimates were obtained for national as well as regional incidences. The four geographical regions were Northeast, Midwest, South, and West as defined by the US Census Bureau in 2000 [26]. Point estimates and 95% CI for incidence rate ratios were calculated using non-Hispanic white as the reference group. Variances for adjusted incidence rates were calculated and regional heterogeneity was tested using ANOVA. Pair wise t-test with Bonferroni correction for multiple comparisons was used to determine statistical differences between pairs of regional incidence rates among APIs and non-Hispanic whites.

### Case fatality

Case fatality, or in-hospital death due to all causes, was calculated for each stroke subtype in non-Hispanic whites, and APIs. The Wilcoxon rank-sum test was used to evaluate race-ethnic differences in age, and the chi-squared test was used to test for differences in stroke subtype. Robust logistic regression [27] was used to calculate the independent association between API race and stroke case fatality. The robust method broadens CI to account for clustering of covariates and outcomes by hospital. In multivariable analyses, we adjusted for patient characteristics such as age, sex, co-morbidity score, length of stay and median income for patient's zip code. Co-morbidity scores were developed using a database version of the Charlson comorbidity index and represent a summary of major secondary diagnoses weighted by severity [28]. We also adjusted for hospital characteristics including geographic region, location (urban vs. rural), teaching status (teaching vs. non-teaching), ownership (government non-federal, private for profit, private non-profit), and size (small, medium, large). To determine whether there was heterogeneity among API populations within the US, we tested for interactions between ethnicity and region by using the likelihood-ratio test [29]. The Stata statistical package was used for all analyses (version 7.0; Stata Corporation, College Station, TX).

## Results

There were 169,386 hospitalizations for stroke in the 1997 NIS database. Of these, 102,268 (60.4%) were for acute ischemic stroke, 5,172 (3.1%) for SAH and 14,907 (8.8%) for ICH. An additional 47,039 (27.7%) were for non-specific stroke diagnoses, with carotid stenosis or occlusion (*ICD-9 *433.1) being the most frequent. Using the average discharge weight for each stroke subtype, we calculated the US projections to be 510,113 cases of acute ischemic stroke, 24,893 cases of SAH, and 73,208 cases of ICH. Most patients were admitted through the emergency department (57.9%). The distribution of race-ethnicity was 80.9% white, 1.6% API, 11.4% African American, 4.7% Hispanic, 0.2% Native American, and 1.3% other. The overall average age was 71.7 ± 13.4 years, and 52.7% were female.

Compared to whites, APIs hospitalized with strokes were younger (Table 1). On average, APIs lived in areas with higher median incomes, were less likely to be discharged to home, had longer hospital stays, and incurred higher total charges. The baseline characteristics for each race-ethnic group were also examined by stroke subtype. The results remained similar to those for all strokes combined in Table 1a, except that APIs with SAH had shorter length of stay than whites. The mean ± SD age for whites were 74.9 ± 12 years for those hospitalized with ischemic stroke, 59.3 ± 16.5 years for SAH, and 72.2 ± 14 years for ICH. APIs were younger across all three stroke subtypes: 71 ± 12.2 years for ischemic stroke, 57.9 ± 18.7 for SAH, and 65 ± 15.3 for ICH. Admissions for ICH made up a larger percentage of total strokes among APIs (17.4%) compared to non-Hispanic whites (8.1%), which could account for some differences in mortality outcomes and age at presentation since patients with ICH tend to be younger [30]. In addition, some of the patient characteristics for API varied significantly across the four regions (Table 2). These include type of hospital, median income for patient's zip code, length of stay, expected payer, and discharge status.

**Table 1:** Characteristics of all hospitalized stroke patients by race-ethnicity.

	**Asian/Pacific Islanders ** Mean ± SD or no.(%) (n = 2,159)	**non-Hispanic Whites ** Mean ± SD or no. (%) (n = 109,632)
Age, years	68.5 ± 14.3	73.3 ± 12.3
Female	47.3	52.1
Region		
Northeast	224 (10.4)	26,471 (24.2)
Midwest	52 (2.4)	21,022 (19.2)
South	186 (8.6)	43,080 (39.3)
West	1697 (78.6)	19,059 (17.4)
		
Urban hospital	2019 (93.5)	93,538 (85.5)
Teaching hospital	821 (38.0)	31,039 (28.4)
		
Type of hospital		
Government, non-fed	432 (20.0)	12,829 (11.7)
Private, not-for-profit	1423 (65.9)	38,748 (72.0)
Private, for-profit	304 (14.1)	17,794 (16.3)
		
Median income for patient's zip code		
$0 – $25,000	254 (12.3)	31,077 (30.3)
$25,001 – $35,000	649 (31.5)	38,748 (37.7)
$35,001 +	1155 (56.1)	32,876 (32.0)
		
Admit from Emergency Dept	1450 (67.4)	58,970 (56.2)
Length of stay, days	8.2 ± 30.4	5.8 ± 19.4
Expected payer		
Medicare	1017 (47.1)	81,956 (74.8)
Medicaid	451 (20.9)	2740 (2.5)
Private	542 (25.1)	21,435 (19.6)
Other	149 (6.9)	3402 (3.1)
Total charges	$24,026 ± $34,928	$14,275 ± $21,039
		
Discharge status		
Routine	789 (36.6)	52,698 (48.1)
Home health care	202 (9.4)	8443 (7.7)
Other facility/AMA*	1130 (43)	39,376 (36.1)
Died	238 (11.0)	8891 (8.1)
Average age at death, years	68.3 ± 14.5	75.5 ± 13.0

* AMA = against medical advice

**Table 2:** Characteristics of all hospitalized Asian patients with stroke by region.

	**Asian/Pacific Islanders ** Mean ± SD or no.(%) (n = 2,159)			
	Northeast (n = 224)	Midwest (n = 52)	South (n = 186)	West (n = 1697)
Age, years	64.3 ± 15.97	64.7 ± 15.7	66 ± 14.8	69.2 ± 14
Female	44.2	40.4	43.6	48.4
Urban hospital	222 (99.1)	46 (88.5)	184 (98.9)	1567 (92.3)
Teaching hospital	160 (71.4)	19 (36.5)	35 (18.8)	607 (35.8)
Type of hospital				
Government, non-federal	64 (28.6)	8 (15.4)	24 (12.9)	336 (19.8)
Private, not-for-profit	155 (69.2)	44 (84.6)	127 (68.3)	1097 (64.6)
Private, for-profit	5 (2.2)	0	35 (18.8)	264 (15.6)
Median income for patient's zip code				
$0 – $25,000	21 (9.6)	27 (52.9)	26 (14.8)	180 (11.2)
$25,001 – $35,000	96 (44.0)	11 (21.6)	36 (20.5)	506 (31.4)
$35,001 +	101 (46.3)	13 (25.5)	114 (64.8)	927 (57.5)
Admit from Emergency Dept	168 (75.3)	34 (72.3)	140 (75.7)	1108 (65.4)
Length of stay, days	16.2 ± 87.5	6.1 ± 7.2	7.8 ± 7.9	8.1 ± 17.3
Expected payer				
Medicare	75 (33.5)	14 (26.9)	78 (41.9)	850 (50.1)
Medicaid	50 (22.3)	17 (32.7)	35 (18.8)	349 (20.6)
Private	65 (29.0)	14 (26.9)	49 (26.3)	414 (24.4)
Other	34 (15.2)	7 (13.5)	24 (12.9)	84 (4.9)
				
Total charges	$25,570 ± $31,001	$14,172 ± $27,282	$15,151 ± $17,994	$25,161 ± $36,920
Discharge status				
Routine	95 (42.8)	24 (46.2)	90 (48.4)	580 (34.2)
Home health	17 (7.7)	7 (13.5)	15 (8.1)	163 (9.6)
Other facility/AMA	82 (36.9)	17 (32.6)	64 (34.4)	765 (45.1)
Died	28 (12.6)	4 (7.7)	17 (9.1)	189 (11.1)
Average age at death, years	69.3 ± 14.4	59.3 ± 23.4	68.6 ± 12.8	68.4 ± 14.5

* AMA = against medical advice

We calculated the national age-adjusted and gender-specific incidence rates for hospitalized acute ischemic stroke, SAH, and ICH. Compared to non-Hispanic whites, APIs were more likely to have SAH and ICH, but rates of acute ischemic stroke were similar (Table 3). There was significant regional variation in incidence rates. API females had higher incidence rate ratios for SAH (1.53, 95% CI 1.41–1.65) and ICH (1.69, 95% CI 1.60–1.79) in the West, but lower rates of stroke everywhere else in the country compared to non-Hispanic whites. Similarly, API males had higher incidence rate ratios for acute ischemic stroke (1.16, 95% CI 1.12–1.21), SAH (1.34, 95% CI 1.12–1.60), and ICH (2.02, 95% CI 1.87–2.18) in the West. In addition, API males also had higher incidence rate ratios for SAH in the Northeast (1.31, 95% CI 1.05–1.63), and ICH in the South (1.21, 95% CI 1.06–1.38) compared to non-Hispanic whites. Regional variation was less dramatic for whites. There was great heterogeneity in incidence rates across regions within each stratum of stroke type (Table 3). In addition, pair-wise t-test calculations revealed that rates for APIs in the West were significantly different from those of all other regions (p < 0.008, after Bonferroni correction).

**Table 3:** Age-adjusted incidence rates and rate ratios of hospitalized stroke stratified by gender for Asians/Pacific Islanders and non-Hispanic whites by stroke subtype and US region.*

	**Incidence Rate per 100,000 (95% CI)**				**Incidence Rate Ratio (95% CI)**	
	**Asians/Pacific Islanders**		**Non-Hispanic Whites**			
	**Female**	**Male**	**Female**	**Male**	**Female**	**Male**
**Ischemic Stroke**						
**National**	111 (102–120)	106 (98–114)	151 (149–153)	131 (129–133)	0.74 (0.72–0.76)	0.81 (0.78–0.83)
Northeast	72 (50–94)	63 (46–80)	187 (183–191)	162 (158–166)	0.39 (0.36–0.41)	0.39 (0.36–0.42)
Midwest	36 (14–58)	35 (18–52)	116 (113–119)	98 (95–101)	0.31 (0.27–0.36)	0.36 (0.31–0.41)
South	79 (55–104)	84 (62–106)	171 (168–170)	158 (155–161)	0.46 (0.43–0.49)	0.53 (0.50–0.57)
West	120 (109–131)	118 (108–128)	128 (125–131)	101 (98–104)	0.94 (0.91–0.98)	1.16 (1.12–1.21)
Heterogeneity	p < 0.001	p < 0.001	p < 0.001	p < 0.001		

**SAH**						
**National**	12 (9–15)	6 (4–8)	8 (7.6–8.4)	5 (4.7–5.3)	1.53 (1.41–1.65)	1.13 (1.00–1.27)
Northeast	5 (0–10)	8 (3–13)	9 (7–10)	6 (5–7)	0.56 (0.41–0.74)	1.31 (1.05–1.63)
Midwest	1 (0–2)	2 (0–5)	7 (6–8)	4 (3–5)	0.09 (0.02–0.23)	0.63 (0.34–1.07)
South	2 (0–4)	1 (0–2)	8 (7–9)	5 (4–6)	0.28 (0.18–0.42)	0.12 (0.04–0.26)
West	16 (12–20)	6 (4–8)	8 (7–9)	5 (4–6)	1.92 (1.73–2.14)	1.34 (1.12–1.60)
Heterogeneity	p < 0.001	p = 0.008	p = 0.079	p < 0.001		

**ICH**						
**National**	25 (21–29)	30 (26–34)	20 (19–21)	19 (18–20)	1.29 (1.22–1.36)	1.58 (1.50–1.67)
Northeast	21 (11–25)	19 (11–27)	25 (24–26)	24 (23–25)	0.86 (0.75–0.98)	0.77 (0.66–0.88)
Midwest	8 (0–18)	12 (2–22)	16 (15–17)	14 (13–15)	0.53 (0.40–0.70)	0.87 (0.68–1.11)
South	20 (10–30)	25 (13–37)	22 (21–23)	21 (20–22)	0.94 (0.82–1.08)	1.21 (1.06–1.38)
West	27 (22–32)	35 (30–40)	19 (18–20)	17 (16–18)	1.42 (1.31–1.54)	2.02 (1.87–2.18)
Heterogeneity	p = 0.039	p = 0.001	p < 0.001	p < 0.001		

* SAH = subarachnoid hemorrhage, ICH = intracerebral hemorrhage. Incidence rate ratio is for Asians/Pacific Islanders vs. non-Hispanic whites. Variances for adjusted rates were calculated and regional heterogeneity was tested using ANOVA.

### Case fatality

Among all stroke hospitalizations, in-hospital death occurred in 14,024 (8.3%). The mean ± SD age for those who died in-hospital was 73.4 ± 14.3 years for all races. APIs (68.3 ± 14.5 years) died at a younger age than non-Hispanic whites (75.5 ± 13 years, p < 0.001). These race-ethnic differences in age at death remained when each stroke subtype was examined separately (data not shown). Case fatality for acute ischemic stroke was 7.3% in white and 6.4% in API (p < 0.001). Case fatality for SAH was 27.5% in white, 29.8% in API (p = 0.081). Case fatality for ICH was 31.9% in white, 26.7% in API (p = 0.002).

We assessed the odds of in-hospital death by stroke subtypes for APIs as compared to whites. In univariate analyses, APIs (OR = 0.78, 95% CI 0.61–0.99, p = 0.05) admitted with ICH were less likely to die in-hospital. However, the association was no longer significant after adjustment for patient and hospital characteristics (age, sex, co-morbidity score, length of stay, and median income for patient's zip code, geographic region, location, teaching status, ownership, and bed size). In addition, there was no interaction between race-ethnicity and region in case fatality (p = 0.50).

## Discussion

Few studies have examined stroke in APIs in the US and no population-based study on stroke in API is available. Thus, the incidence of stroke in API in the US is not known. Using a 1997 national database to overcome problems with small sample size, we found that the overall US incidence rates for SAH and ICH were higher for APIs than for non-Hispanic whites. Our age-adjusted and gender-specific incidence rates for total stroke and for each stroke subtype for non-Hispanic whites are similar to those reported elsewhere[31,32]. The age and gender-adjusted incidence of first-ever strokes among whites in Rochester was 179 per 100,000[31]. A recent population-based study of the greater Cincinnati metropolitan area reported the age and gender-adjusted incidence rates for non-Hispanic whites to be 212 per 100,000 for first and recurrent strokes, 137 per 100,000 for first ischemic stroke, 6 per 100,000 for first SAH, and 18 per 100,000 for first ICH[32].

We found a significant interaction between race-ethnicity and geographic region. The stroke incidence rates varied over 3-fold in APIs among different US regions. Geographic variation was less dramatic in non-Hispanic whites. It is not likely that the heterogeneity in rates was due to small numbers given that the incidence rates were statistically heterogeneous among the regions for all stroke types. This dramatic regional variation might reflect the different distributions of subgroups of APIs (such as Chinese, Japanese, Filipinos, etc.) in the US, as suggested by the significant differences in patient characteristics across regions (Table 2). Striking regional differences in stroke incidence between northern and southern China [3], and between countries in Asia[7] have been reported. These reports and our current findings suggest that combining these distinct subpopulations into one race-ethnic category may be inappropriate.

Our finding of a higher incidence of ICH in APIs is consistent with prior reports from Asia of high rates of hemorrhagic strokes in Asians [3,5,33]. Studies from Asia also reveal a high prevalence of hypertension in these populations [33]. It is not known how much uncontrolled hypertension contributes to the differential burden of hemorrhage in APIs in the US. Other differences in diet (such as a higher salt intake), prevalence of smoking, or genetic factors might also contribute to this difference in ICH incidences.

On average, APIs had longer hospital stays for ischemic stroke and ICH, had higher total hospital charges, and were more likely to be discharged to another facility rather than home. These findings were not explained by differences in stroke subtypes. One possible explanation of these findings is that APIs are hospitalized with more severe strokes, as has been suggested by one prior study showing higher case fatality rates for APIs with ICH [12]. However, we found no significant difference in all-cause in-hospital mortality between API and non-Hispanic white after adjusting for important potential confounders. Whether cultural differences in preferences for end-of-life care could mask an impact of greater stroke severity on case fatality cannot be answered in our study.

There are several limitations to our study. We only included in our analyses patients who were hospitalized for stroke. Thus, non-hospitalized strokes, which account for approximately 10% of all strokes in the US [34], were not included. Furthermore, incidence-rate calculations were based on all stroke hospitalizations including recurrent cases. The accuracy of our incidence estimates depends on the accuracy of *ICD-9 *coding in the database. Although we used only *ICD-9 *codes that were previously identified as more accurate in order to enhance the precision of our estimates, reliability of coding is still imperfect [21-23]. In addition, by using only *ICD-9 *codes in the primary position, we recognize that we increase specificity but decrease the sensitivity of case ascertainment [35]. Another limitation is the lack of reliable coding for comorbidities. Consequently, we were unable to fully adjust for severity of disease or to evaluate risk factors. We also did not have data on do-not-resuscitate status of the patients, which could influence the in-hospital mortality rate. Furthermore, API was not broken down into distinct ethnic subgroups in the database. This is a problem encountered by all similar studies. Despite the limitations, the study reveals several important and interesting findings that deserve further exploration.

## Conclusion

In summary, analyses from a large nationwide database revealed that APIs appear to have a higher risk than non-Hispanic whites for ICH and SAH. However, differences between APIs and whites vary dramatically between regions, with the highest rates among APIs in the West. Which sub-populations of APIs are at greater risk, and the factors that predict this elevated risk remain to be determined. APIs come from nearly 50 distinct countries and ethnic groups, each with its own set of cultures and traditions, and potentially very different genetic and/or environmental risk factors for stroke. Our findings suggest the need for further studies to examine the differences in stroke incidence and risk factors among the subpopulations of APIs. Furthermore, understanding of the racial-ethnic differences in stroke should lead to the development of more efficient and effective stroke prevention strategies.

## Competing interests

The author(s) declare that they have no competing interests.

## Authors' contributions

MNH and SCJ both contributed to the conception and design of the study. MNH conducted the analyses of the data and drafted the manuscript. Both authors made substantial contributions to the revision of the manuscript, and approved the final version.

## Pre-publication history

The pre-publication history for this paper can be accessed here:


